# Is the red fox (*Vulpes vulpes*) a competent definitive host for *Taenia multiceps*?

**DOI:** 10.1186/s13071-015-1096-7

**Published:** 2015-09-25

**Authors:** Antonio Varcasia, Claudia Tamponi, Gabriele Tosciri, Anna Paola Pipia, Francesco Dore, Rolf Karl Schuster, Omnia Mohamed Kandil, Maria Lucia Manunta, Antonio Scala

**Affiliations:** Laboratorio di Parassitologia, Ospedale Didattico Veterinario, Dipartimento di Medicina Veterinaria, Università degli Studi di Sassari, Sassari, Italy; Central Veterinary Research Laboratory, Dubai, United Arab Emirates; Department of Parasitology and Animal Diseases, National Research Centre, El-Behouse Street, Dokki, P.O. Box 12622, Cairo, Egypt

**Keywords:** Red fox, *Vulpes vulpes*, *Taenia multiceps*, Coenurosis, Sheep, Goat

## Abstract

**Background:**

Shepherd and stray dogs are thought to represent the primary definitive hosts of Coenurosis by *Taenia multiceps*, due to their feeding habits which translate into high chances of coming into contact with infected intermediate hosts. Nonetheless, little attention has been paid to the role of the red fox (*Vulpes vulpes*) in the epidemiology of coenurosis. In fact a knowledge gap exists on the role played by red foxes in the epidemiology of *Taenia multiceps* and the capability of this parasite to produce fertile and viable eggs in this wild canid, i.e. on the occurrence of a sylvatic cycle.

This study investigates the role of the red fox (*Vulpes vulpes*) in the epidemiology of *T. multiceps* and related metacestodoses.

**Methods:**

The small intestine of 63 red foxes was macroscopically examined for the presence of cestodes. Adult parasites were identified morphologically as being *T. multiceps*. Tapeworm eggs were counted and stored at 4 °C in physiological saline solution prior to experimental infection of four sheep and one goat. Sheep were inoculated orally on Day 0 with 3000 (sheep 1), 5000 (sheep 2 and 3) or 7000 eggs (sheep 4), while the goat was infected with 5000 eggs of *T. multiceps.* The animals were followed-up regularly by MRI and underwent surgical treatment between days 180 to day 240 post infection. Collected coenuri were identified using morphological and molecular methods.

**Results:**

A total of 6.3 % of red foxes were found infected with *T. multiceps* and the eggs obtained from the worms were determined to have a viability of 45.4 %. Two of the challenged sheep and the goat developed disease compatible with *T. multiceps*. Morphometrical features of the cysts were consistent with those of *T. multiceps*; nucleotide amplification and sequencing of mitochondrial genes (i.e., *cox*1 and Nd1) from the metacestode material confirmed the identification.

**Conclusions:**

The present study is the first to provide evidence of the role of the red fox as a competent definitive host for *T. multiceps,* thus changing the epidemiological scenarios of infections by this cestode.

## Background

Coenurosis or “Gid”, known previously as Coenurus cerebralis, is a parasitic disease caused by the metacestode stage of *Taenia multiceps* (Cestoda, Teniidae). The adult tapeworm inhabits the small intestine of a number of domestic and wild carnivores, including dogs, jackals, foxes and coyotes [1]. Eggs of *T. multiceps* are excreted in the environment with the faeces of the definitive hosts and ingested by intermediate herbivore hosts including sheep, goats, horses, cattle, camels, deer and pigs [2, 3]. Following the ingestion of the eggs, the oncosphere hatches, burrows its way through the intestinal wall and reaches the central nervous system (CNS) and other organs *via* the bloodstream [4]. The infection is often lethal in intermediate hosts and the parasite is a significant cause of economic losses in many areas of the world [5].

Coenurosis has been documented in scattered foci in the Americas and parts of Europe, Asia and Africa, although its distribution is most likely global, with the exception of Australia [6]. The infection is more common in temperate regions, such as the Mediterranean basin and Sardinia Island, where different intraspecific genetic variants have been identified [7–9]. In particular, a genetic variant of this parasite (i.e. Tm4) is also responsible for non-cerebral coenurosis in goats, where the parasite invades the subcutaneous fascia, peritoneal areas, and intramuscular tissues [10–14].

Coenurosis is a zoonotic disease in which humans act as accidental hosts and may develop different forms of neurological diseases. Over 100 human cases have been described from different countries including Italy, Egypt and the United States [6].

Shepherd and stray dogs are thought to represent the primary definitive hosts of this metacestodosis, due to their feeding habits, which translate into high chances of coming into contact with infected intermediate hosts [5]. Nonetheless, little attention has been paid to the role of the red fox (*Vulpes vulpes*) in the epidemiology of coenurosis. Several authors have reported *T. multiceps* from fox species, but with prevalences usually under 1 % [15–18]. A survey in the Tartar Republic of Russia identified 28.2 % positivity for *T. multiceps* in necropsied foxes (*n* = 350) [19]. Other descriptions of *T. multiceps* in red foxes originated from Germany (3.3 %) [20], Perù (2 %) [21], Jordan (3.8 %) [22], Iran (4.8 and 8.2 %) [23, 24] and China (6 % in Tibetan sand foxes) [25]. To date, *T. multiceps* has never been described from red foxes of Sardinia, Italy [26, 27], where sheep coenurosis is endemic.

A knowledge gap exists on the role played by red foxes in the epidemiology of *T. multiceps* and the capability of this parasite to produce fertile and viable eggs in the intestine of red foxes, i.e. on the occurrence of an alternative lifecycle.

Therefore, in this study, the role of the red fox as a competent definitive host for *T. multiceps* was investigated to better understand the epidemiology of this important taeniid and of the metacestodosis it causes.

## Ethics

This study was approved by the National Research Centre (NRC) – Medical Research Ethics Committee (MREC) on animal ethics, Registration number 14099 of 14/09/2014 and executed following the recommendations of European Council Directive (86/609/EEC) on the protection of animals used for experimental purposes.

## Methods

Between 2012 and 2014, 63 carcasses of red foxes (*Vulpes vulpes*), road-killed or legally hunted in Sardinia Island (Western Mediterranean, Italy - 40° 00′ 00″ N; 9° 00′ 00″ E), were referred to the Laboratory of Parasitology of the Veterinary Teaching Hospital in Sassari, for necropsy. At necropsy, the small intestine was macroscopically examined and dissected under a stereomicroscope for detection of cestodes. Parasites collected were washed in saline solution and the gravid segments were collected and stored at 4 °C in TCM 199 (Minitüb GmbH, Tiefenbach, Germany). Cestode specimens were individually mounted on glass slides and morphologically identified using morphometrical keys [28, 29]. A section of each parasite was stored for molecular identification using protocols described elsewhere [7, 30].

Eggs were separated from gravid proglottids using a surgical blade, counted in a McMaster slide and stored at 4 °C in physiological saline solution prior to experimental infections. Egg vitality tests were performed according to the methodology described by Deplazes *et al.* [31]. Briefly, 0.3 ml of Sodium Hypochlorite (SH) solution (2 % active chlorine, pH 12) was added to 0.4 ml egg suspension (2500 eggs/ml). Within 1 min (i.e. before destruction of embryophores occurred), the total number of eggs was determined in a McMaster-chamber. Four to 5 min later, oncospheres with intact membranes were counted again. SH resistance was calculated from triplicate counts as percentage of intact oncospheres. Eggs were subsequently diluted in PBS, and a batch of eggs (5000/ml) was prepared for the challenge in the intermediate hosts.

Four 16-week old male sheep and one goat, kept indoors since birth, were dewormed prior to the trial (4 ml /40 kg b.w. of Albendazole 1.9 %; Valbazen, Zoetis). The egg suspension was enclosed into capsules resistant to gastric digestion (Sansyoiyaku, Shizuoka, Japan) and animals were orally inoculated using a rumen bolus applicator at Day 0 (D0) with 3000 (Sheep 1), 5000 (Sheep 2 and Sheep 3) and 7000 eggs (Sheep 4), while the goat was infected with 5000 eggs of *T. multiceps*.

Following experimental infection, animals were housed under usual conditions with free access to food and water. Animals were checked daily to detect clinical signs and behavioural changes (i.e., temperature, physiologic functions, symptoms related to coenurosis or other diseases). A neurological examination and magnetic resonance imaging (MRI) scan according to Manunta *et al.* [32] was performed for each animal every 30 days, and the size, localization and number of coenuri detected were documented. Surgical treatment of positive animals was performed at the end of the challenge.

The coenuri collected at surgery were examined under a light microscope without staining, in order to assess viability, number and size of clusters of protoscoleces, hook size and numbers. Microscopical images and measurements were acquired using a digital image processing system (LC micro Image Acquisition Software V.5.2, Olympus). Aliquots of protoscoleces were used for molecular identification [7].

## Results

**Fig. 1:**
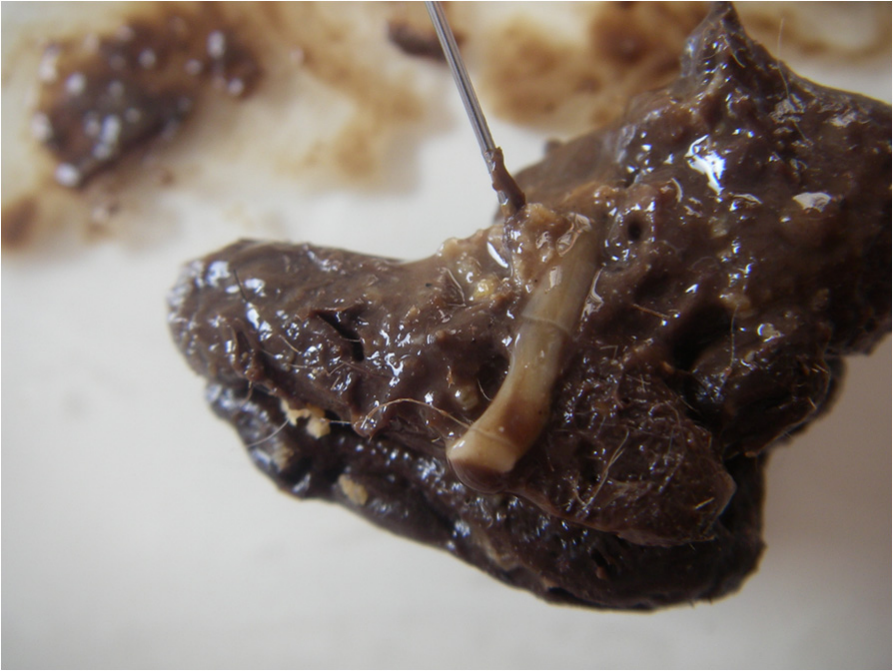
Gravid proglottids of *Taenia multiceps* recovered in the rectum of a necropsied fox

**Fig. 2:**
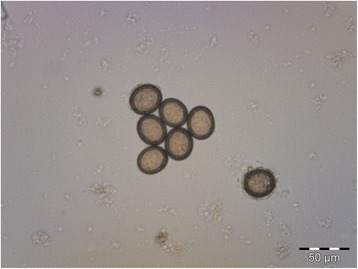
Eggs of *Taenia multiceps* obtained from gravid proglottids (200× magnification)

Four (6.3 %) out of the 63 examined red foxes were infected by *T. multiceps* (Figs. 1 and 2). The percentage of intact oncospheres at the viability test was 45.4 % (567.7/1172 in 0.4 ml of egg suspension).

**Fig. 3:**
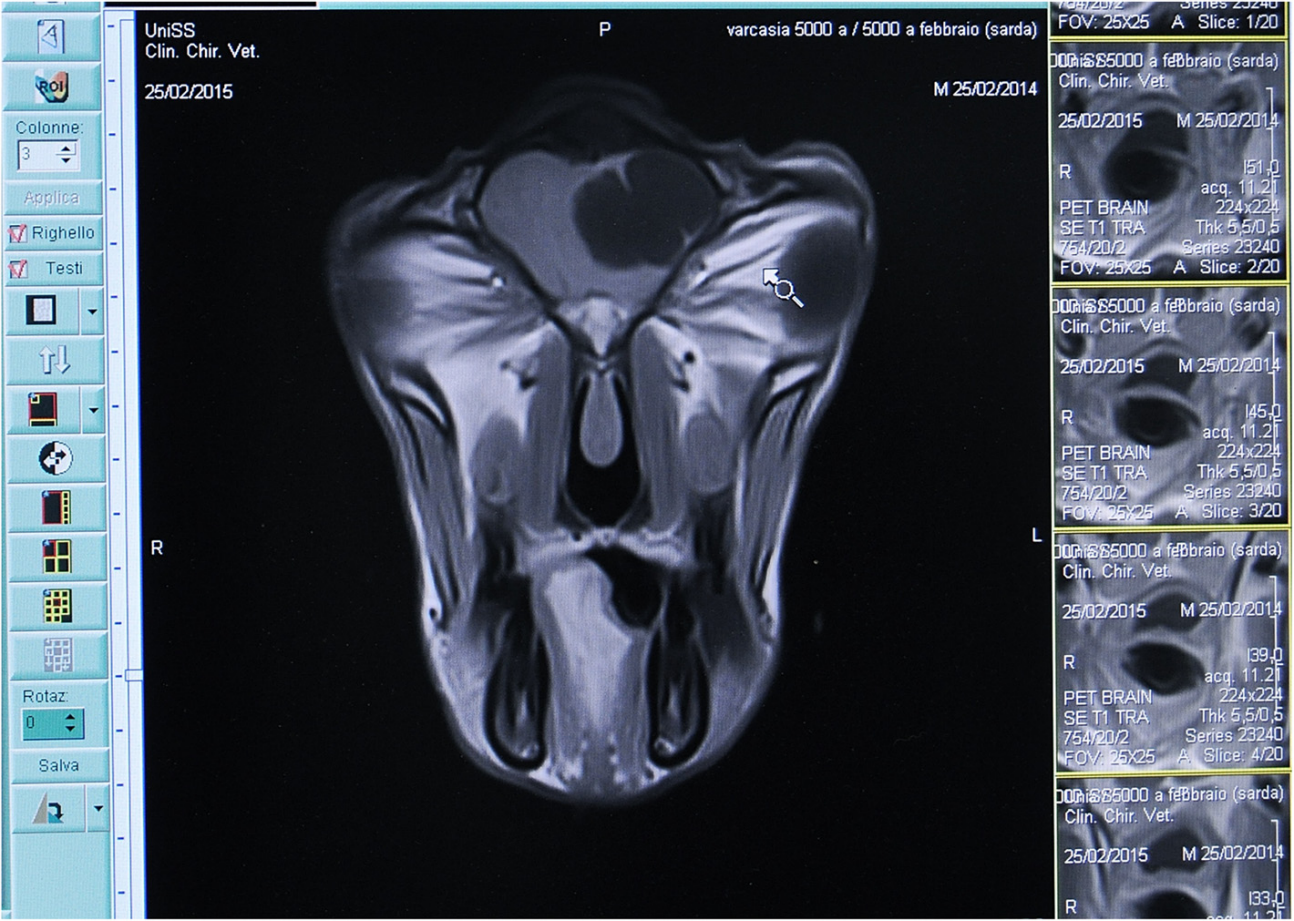
Screenshot of magnetic resonance imaging (MRI) scan of a sheep challenged with 5000 *Taenia multiceps* eggs

Three of the animals challenged with *T. multiceps* eggs developed coenurosis as assessed by MRI (Fig. 3) (Table 1) at 30 days. Two sheep developed one coenurus each while sheep 4 and the goat developed 4 and 3 coenuri, respectively.

**Table 1:** Animals, challenge doses of *Taenia multiceps* eggs and number and location of coenuri found

Animal ID	Infection dose (eggs)	Number of Coenuri	Location	Viability	Strain	Surgery day	Recovery
Sheep 1	3000	0	–	–	–	–	–
Sheep 2	5000	1	Brain	Viable	Tm1	Day 284	Complete
Sheep 3	5000	0	–	–	–	–	–
Sheep 4	7000	4	Brain	Viable	Tm1	Day 375	Complete
Goat 1	5000	3	Brain	Viable	Tm1	Day 226	Complete

**Fig. 4:**
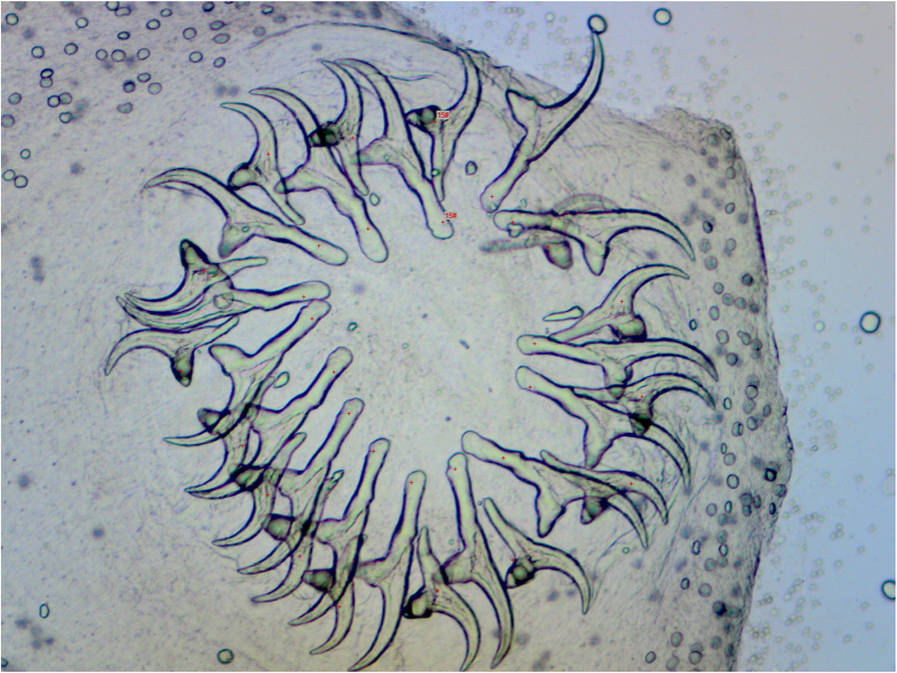
Light microscopy, hooks of a protoscolex (50× magnification)

Following surgical procedure for the removal of the coenuri (day 180–240), all animals fully recovered. All recovered metacestodes were viable, with visible protoscoleces. The size of the cysts as well the number of clusters of protoscoleces, their size and the morphometric data of the hooks (Fig. 4) were consistent with *T. multiceps* cysts (Table 2). Molecular analyses of both adult tapeworms and metacestodes displayed 100 % nucleotide identity to *T. multiceps* Tm1 strain [Genbank accession numbers: AY669089.2; DQ309767.1] [7].

**Table 2:** Features and size of recovered coenuri in animals challenged with *Taenia multiceps* eggs

Animals	Location	Size (mm)	Clusters	Protoscolices size (μm)	Scolex size (μm)	Suckers size diameter (μm)	Large hooks		Small hooks	
							Number	Size (μm)	Number	Size (μm)
Sheep 2	1 (left frontal lobe)	25 × 15	6	1428 – 1627	284 – 363	236 – 358	15	170 – 173	15	113 – 116
Sheep 4	1 (left parietal lobe)	15 × 20	2	1447 – 1804	266 – 352	220 – 330	14	144 – 163	14	122 – 124
Sheep 4	2 (left side falx cerebri)	27 × 24	5	1193 – 1720	247 – 291	208 – 280	15	158 – 160	15	108 – 115
Sheep 4	3 (right temporal lobe)	30 × 35	6	1035 – 1837	304 – 394	271 – 317	13	146 – 149	13	104 – 108
Sheep 4	4 (right frontal lobe)	20 × 22	5	1062 – 1541	289 – 431	297 – 362	14	164 – 171	15	113 – 115
Goat 1	1 (right temporal lobe)	20 × 15	7	1001 – 1639	242 – 274	196 – 240	16	117 – 119	16	97 – 102
Goat 1	2 (left side cerebellum)	72 × 48	2	1028 – 1586	228 – 334	275 – 298	15	153 – 157	15	106 – 107
Goat 1	3 (4th ventricle)	20 × 18	5	977 – 2092	249 – 330	286 – 370	16	161 – 167	16	110 – 118

## Discussion

The present study provides experimental evidence of the role of the red fox as a competent definitive host for *T. multiceps*, clearly indicating that coenurosis by *T. multiceps* can be maintained and spread by wild canids.

In spite of previous speculations hypothesising a possible role for the red fox in transmitting *T. multiceps*, this is the first study providing unequivocal data showing that foxes excrete proglottids with viable and infective eggs that could therefore maintain the parasite lifecycle.

A similar issue has surrounded another important cestode, *Echinococcus granulosus*, with some authors claiming that parasites infecting red foxes were unable to produce fertile eggs [26, 33] and others reporting an active role for this canid as a valid definitive host for this cestode [34].

In the past, several authors have also questioned the role of the red fox as a definitive host for *T. multiceps*, because of the presumptive inability of this carnivore to access the sheep CNS through the skull [35]. However, it has been reported that the presence of cysts is associated with thinner skull bones, thus facilitating access, even to foxes [6]; the first author of the present paper has personally witnessed foxes accessing the full content of a sheep skull (CNS and eventually coenuri) *via* the *foramen magnum* without breaking the skull itself [Varcasia A., personal observations].

The red fox is the most widespread wild member of the Carnivora, distributed from the Arctic Circle to subtropical regions in North America and Eurasia as well as in in North Africa. Its distribution range has increased alongside human expansion, and it now includes Australia. Red fox populations are increasing in numbers and their expansion towards urban areas has been reported over the last two decades. This is considered to have contributed to the enlarging distribution of *Echinococcus multilocularis* (EM) and alveolar echinococcosis cases in urban areas of Europe and North America [36].

Home ranges of red foxes in the United States span from 2. to 19.9 km^2^, ~50 km^2^ in Iran, from 4 to 12 km^2^ in Sweden, and from 0.21 (urban) to 10.0 (rural) km^2^ in England, with males occupying larger home ranges than females [37].

For this reason the role of foxes in the epidemiology of coenurosis must not be underestimated. During the mating period in spring, males are able to travel several kilometres, both for mating and hunting. This time span also coincides with the period in which replacement sheep (3–4 months animals) are usually taken out at pasture for the first time in many Mediterranean locations. This period is considered to be crucial for infection of replacement sheep [8, 9].

Control of *T. multiceps* transmission by foxes is bound to be challenging, given the large home ranges and the scary and suspicious attitude of this species. The sanitary education of the farmers and the prophylactic measures (e.g., concerning the proper disposal of dead/ butchered animals) are of primary importance in the prevention of the disease and to reduce the risk of infection of foxes in areas where coenurosis is endemic. One of the first steps that should be implemented in these areas is represented by mandatory slaughtering of *T. multiceps* infected animals at diagnosis, to avoid contact between infected carcasses and dogs and foxes in the field, as well as appropriate disposal of skull and offal of slaughtered animals [5].

In endemic areas for *E. multilocularis*, the use of praziquantel baits every 1–6 km has been effective in reducing the cestode parasite pressure in foxes. This approach is also cost effective if implemented for several decades in restricted, most relevant areas for the transmission of the parasites [36]. For *T. multiceps*, the use of Praziquantel baits could be useful particularly in inaccessible and wild areas where the disease is endemic, as well as in proximity of urbanized areas. Vaccination of intermediate hosts (with combinations of parasite antigens expressed in recombinant form), and in particular of replacement animals, has been shown to be effective and could represent an alternative control method for coenurosis, together with other metacestodoses such as cystic echinococcosis [8, 9].

According to Verster and Tustin [38], the number of eggs necessary to develop Coenurosis in sheep is in excess of 5000 eggs; the results of our experiments, even conducted on a limited number of animals, are consistent with this finding.

Two of the challenged animals did not develop Coenurosis; this could be related to some animals being refractory to the infection, for instance due to genetic factors or immune responses to the parasite [8].

The Coenuri detected in the challenged animals displayed morphological features and size typical of *T. multiceps*. Molecular identification confirmed the diagnosis and revealed that all the cysts removed belonged to the genetic variant Tm1, which represents the most common genotype detected in intermediate hosts in Sardinia as well as in other geographical areas [7, 13].

Considering the genetic variant involved in this report and the number of cysts recovered in all challenged animals, it seems that the number of coenuri in infected animals should be more related to the quantity and viability of eggs ingested rather than to a particular strain (ie. Tm1/Tm3), as had been speculated in the past [7].

## Conclusions

Based on the findings of the present study, we argue that the red fox should be recognised as an alternative definitive host of *T. multiceps* and, considering that this species is becoming increasingly widespread in several European countries, the role of this carnivore must be taken into account when planning control strategies for the prevention of this metacestodosis in livestock, as well as public health-related risks.

## References

[1] Rostami S, Beech RN, Salavati R, Baneshi MR, Kamyabi H, Harandi MF (2013). Morphometric analysis of larval rostellar hooks in *Taenia multiceps* of sheep in Iran and its association with mitochondrial gene variability. Iran J Parasitol.

[2] Scala A, Cancedda GM, Varcasia A, Ligios C, Garippa G, Genchi C (2007). A survey of *Taenia multiceps* coenurosis in Sardinian sheep. Vet Parasitol.

[3] Varcasia A, Pipia AP, Arru D, Pes AM, Tamponi C, Dore F, Garippa G, Scala A (2013). Morphological and molecular characterization of bovine coenurosis in Sardinia, Italy. Parasitol Res.

[4] Paltrinieri S, Varcasia A, Cazzaniga S, Giordano A, Pipia AP, Marrosu R, Scala A (2010). Brain creatine kinase isoenzyme (CK-BB) as a possible biomarker for the diagnosis *in vivo* of ovine coenurosis in a naturally infected flock. Small Ruminant Res.

[5] Varcasia A, Tanda B, Giobbe M, Solinas C, Pipia AP, Malgor R, Carmona C, Garippa G, Scala A (2011). Cystic echinococcosis in Sardinia: farmers’ knowledge and dog infection in sheep farms. Vet Parasitol.

[6] Scala A, Varcasia A (2006). Updates on morphobiology, epidemiology and molecular characterization of coenurosis in sheep. Parassitologia.

[7] Varcasia A, Lightowlers MW, Cattoli G, Cancedda GM, Canu S, Garippa G, Scala A (2006). Genetic variation within *Taenia multiceps* in Sardinia, Western Mediterranean (Italy). Parasitol Res.

[8] Gauci C, Vural G, Oncel T, Varcasia A, Damian V, Kyngdon CT, Craig PS, Anderson GA, Lightowlers MW (2008). Vaccination with recombinant oncosphere antigens reduces the susceptibility of sheep to infection with *Taenia multiceps*. Int J Parasitol.

[9] Varcasia A, Tosciri G, Coccone GN, Pipia AP, Garippa G, Scala A, Damien V, Vural G, Gauci CG, Lightowlers MW (2009). Preliminary field trial of a vaccine against coenurosis caused by *Taenia multiceps*. Vet Parasitol.

[10] Oryan A, Nazifi S, Sharifiyazdi H, Ahmadnia S (2010). Pathological, molecular, and biochemical characterization of *Coenurus gaigeri* in Iranian native goats. J Parasitol.

[11] Schuster RK, Sivakumar S, Wieckowsky T (2010). Non-cerebral coenurosis in goats. Parasitol Res.

[12] Varcasia A, Jia WZ, Yan HB, Manunta ML, Pipia AP, Garippa G, Scala A, Schuster RK (2012). Molecular characterization of subcutaneous and muscular coenurosis of goats in United Arab Emirates. Vet Parasitol.

[13] Oryan A, Akbari M, Moazeni M, Amrabadi OR (2014). Cerebral and non-cerebral coenurosis in small ruminants. Trop Biomed.

[14] Akbari M, Moazeni M, Oryan A, Sharifiyazdi H, Amrabadi O. Experimental cerebral and non-cerebral coenurosis in goats: A comparative study on the morphological and molecular characteristics of the parasite. Vet Parasitol. 2015; (*In press*). 10.1016/j.vetpar.2015.06.013 26116455

[15] Shaldybin LS (1950). Helminth fauna of hunting animals in the Mordova state reserve (In Russian).

[16] Kadenacii AN. Helminth fauna of mammals of Crimea and trial to control major helminthoses in farm animals (In Russian) (source not given), Omsk: 1957;124. cited in Bondareva V.I. Coenurus invasion in domestic and wild animals. Academy of Sciences of the Kazakh 1963, SSR publishing house, Almaty, 355 pp.

[17] Igrashev (1958). On the helminth fauna of domestic and wild animals in Samarkand region (In Russian). Uzbekistan Biol J.

[18] Muminov P Ch. On the helminth fauna of of foxes in Uzbekistan (In Russian). Theses of papers of the scientific conference of Allunion Helminthological Society 1962 (part 2). Moscow: Academy of Sciences. cited in Bondareva VI: Coenurus invasion in domestic and wild animals. Academy of Sciences of the Kazakh 1963, SSR publishing house, Almaty, 355 pp.

[19] Troickaja AA. Materials on the helminth fauna of foxes in the Tartar Autonomous Sovjet Republic (In Russian). Papers of the Allunion Scientific Institute of Hunting Industry 1955; 14. Cited in Bondareva VI: Coenurus invasion in domestic and wild animals. Academy of Sciences of the Kazakh 1963, SSR publishing house, Almaty, 355 pp.

[20] Ballek D, Takla M, Ising-Volmer S, Stoye M (1992). The helminth fauna of red foxes (Vulpes vulpes Linnaeus 1758) in north Hesse and east Westphalia. 1. Cestodes. Dtsch Tierarztl Wochenschr.

[21] Moro PL, Ballarta J, Gilman RH, Leguia G, Rojas M, Montes G (1998). Intestinal parasites of the grey fox (*Pseudalopex culpaeus*) in the central Peruvian Andes. J Helminthol.

[22] El-Shehabi FS, Abdel-Hafez SK, Kamhawi SA (1999). Prevalence of intestinal helminths of dogs and foxes from Jordan. Parasitol Res.

[23] Dalimi A, Sattari A, Motamedi G (2006). A study on intestinal helminthes of dogs, foxes and jackals in the western part of Iran. Vet Parasitol.

[24] Nabavi R, Manouchehri Naeini K, Zebardast N, Hashemi H (2014). Epidemiological study of gastrointestinal helminthes of canids in Chaharmahal and Bakhtiari province of Iran. Iran J Parasitol.

[25] Li W, Guo Z, Duo H, Fu Y, Peng M, Shen X, Tsukada H, Irie T, Nasu T, Horii Y, Nonaka N (2013). Survey on helminths in the small intestine of wild foxes in Qinghai, China. J Vet Med.

[26] Deaina S, Arru E (1962). *Echinococcus granulosus* in *Vulpes vulpes* della Sardegna. Parassitologia.

[27] Leoni A, Garippa G, Masala S (1986). Osservazioni sull’elmintofauna della volpe (*Vulpes vulpes*) in Sardegna. Parassitologia.

[28] Verster A (1969). A taxonomic revision of the genus *Taenia* Linnaeus, 1758 s.str. Onderstepoort J Vet Res.

[29] Loos-Frank B (2000). An update of Versters (1969): ‘Taxonomic revision of the genus *Taenia* Linnaeus’ (Cestoda) in table format. Syst Parasitol.

[30] Otranto D, Varcasia A, Solinas C, Scala A, Brianti E, Dantas-Torres F, Annoscia G, Martin C, Mutafchiev Y, Bain O (2013). Redescription of *Cercopithifilaria bainae* Almeida & Vicente, 1984 (Spirurida, Onchocercidae) from a dog in Sardinia, Italy. Parasit Vectors.

[31] Deplazes P, Grimm F, Sydler T, Tanner I, Kapel CMO (2005). Experimental alveolar echinococcosis in pigs, lesion development and serological follow up. Vet Parasitol.

[32] Manunta ML, Evangelisti MA, Burrai GP, Columbano N, Ligios C, Varcasia A, Scala A, Sanna Passino E (2012). Magnetic resonance imaging of the brain and skull of sheep with cerebral coenurosis. Am J Vet Res.

[33] Gemmell MA. Hydatid disease in Australia.VI. Observations on the carnivora of New South Wales as definitive hosts of Echinococcus granulosus (Batsch, 17E6) (Rudolphi, 1801), and their role in the spread of hydatidiasis in domestic animals. Aust Vet J. 1959;35:450–55.

[34] Thompson RCA (1983). The susceptibility of the European red fox (*Vulpes vulpes*) to infection with *Echinococcus granulosus* of Australian sheep origin. Ann Trop Med Parasitol.

[35] Deiana S (1971). Stato attuale della diffusione della Cenurosi cerebrale negli ovini e nell’uomo in Sardegna. Parassitologia.

[36] Hegglin D, Deplazes P (2013). Control of *Echinococcus multilocularis*: strategies, feasibility and cost-benefit analyses. Int J Parasitol.

[37] Rountree GH. Comparative study of the home range and habitat usage of red foxes and gray foxes in an urban setting: a preliminary report. In: Shaw WW, Harris LK & Van Druff L (Eds.) 2004;238–244. Proceedings of the 4th International Symposium on Urban Wildlife Conservation, May 1–5, 1999. Tucson, Arizona, 368 pp.

[38] Verster A, Tustin RC (1982). Treatment of the larval stage of *Taenia multiceps* with praziquantel. J S Afr Vet Assoc.

